# Fecal microbiota transplantation in Parkinson's disease—A randomized repeat-dose, placebo-controlled clinical pilot study

**DOI:** 10.3389/fneur.2023.1104759

**Published:** 2023-03-02

**Authors:** Herbert L. DuPont, Jessika Suescun, Zhi-Dong Jiang, Eric L. Brown, Heather T. Essigmann, Ashley S. Alexander, Andrew W. DuPont, Tehseen Iqbal, Netanya S. Utay, Michael Newmark, Mya C. Schiess

**Affiliations:** ^1^Microbiome Research Center, Kelsey Research Foundation, Houston, TX, United States; ^2^Division of Epidemiology, Human Genetics and Environmental Sciences, University of Texas School of Public Health, Houston, TX, United States; ^3^Department of Internal Medicine, University of Texas McGovern Medical School, Houston, TX, United States; ^4^Medical Services and Specialties, Kelsey Seybold Clinic, Houston, TX, United States; ^5^Department of Neurology, University of Texas McGovern Medical School, Houston, TX, United States; ^6^Department of Internal Medicine, University of Texas Southwestern Medical Center, Dallas, TX, United States

**Keywords:** Parkinson's disease, fecal microbiota transplantation, microbiome, dysbiosis, constipation

## Abstract

**Background and purpose:**

The intestinal microbiome plays a primary role in the pathogenesis of neurodegenerative disorders and may provide an opportunity for disease modification. We performed a pilot clinical study looking at the safety of fecal microbiota transplantation (FMT), its effect on the microbiome, and improvement of symptoms in Parkinson's disease.

**Methods:**

This was a randomized, double-blind placebo-controlled pilot study, wherein orally administered lyophilized FMT product or matching placebo was given to 12 subjects with mild to moderate Parkinson's disease with constipation twice weekly for 12 weeks. Subjects were followed for safety and clinical improvement for 9 additional months (total study duration 12 months).

**Results:**

Fecal microbiota transplantation caused non-severe transient upper gastrointestinal symptoms. One subject receiving FMT was diagnosed with unrelated metastatic cancer and was removed from the trial. Beta diversity (taxa) of the microbiome, was similar comparing placebo and FMT groups at baseline, however, for subjects randomized to FMT, it increased significantly at 6 weeks (*p* = 0.008) and 13 weeks (*p* = 0.0008). After treatment with FMT, proportions of selective families within the phylum Firmicutes increased significantly, while proportion of microbiota belonging to Proteobacteria were significantly reduced. Objective motor findings showed only temporary improvement while subjective symptom improvements were reported compared to baseline in the group receiving FMT. Constipation, gut transient times (NS), and gut motility index (*p* = 0.0374) were improved in the FMT group.

**Conclusions:**

Subjects with Parkinson's disease tolerated multi-dose-FMT, and experienced increased diversity of the intestinal microbiome that was associated with reduction in constipation and improved gut transit and intestinal motility. Fecal microbiota transplantation administration improved subjective motor and non-motor symptoms.

**Clinical trial registration:**

ClinicalTrial.gov, identifier: NCT03671785.

## Introduction

Dysregulation of the microbiome–gut–brain axis is a crucial pathoetiology preceding motor symptoms in Parkinson's disease. A working hypothesis is that the alpha-synuclein aggregates spread in a prion-like fashion from the gut to the brainstem substantia nigra (SN) and cortex via the vagus and glossopharyngeal nerves (1). Parkinson's disease-associated alterations of the gut microbiome predict disease-relevant changes significant in metabolic functions (2) and disease progression (3). Parkinson's medications can also have important effects on the intestinal microbiome (4).

In a mouse model of Parkinson's disease, fecal microbiota transplantation (FMT) reduced gut microbiota alterations and decreased inflammation by activating microglia and astrocytes in the SN (5). In another study of MPTP-induced Parkinson's disease in mice, FMT reduced symptoms, decreased expression of alpha-synuclein, inhibited microglia activation, and blocked TLR4/P13K/AKT/NF-_K_B signaling in the SN (6).

In the present pilot study, we sought to determine the safety and tolerability of multiple doses of fecal microbiota given to subjects with mild to moderate Parkinson's disease. Additionally, we hoped to (a) show changes in the intestinal microbiome with multiple doses of FMT; (b) to determine FMT effects on constipation; and (c) collect preliminary data on other effects in Parkinson's disease. Fecal microbiota transplantation has been examined in a variety of medical disorders associated with altered intestinal microbiomes, where increases in post-treatment diversity of colonic microbiota were frequently associated with improvements in health (7).

## Materials and methods

### Patients

Male and female patients 55–80 years of age, with a history of constipation and mild to moderate Parkinson's disease were recruited for the study conducted between June 20, 2019, to May 30, 2020. Enrollment criteria included a diagnosis of Parkinson's disease by UK Brain Bank criteria (8); robust response to dopaminergic therapy defined as ≥33% reduction of the Unified Parkinson's Disease Rating Scale (UPDRS) motor score in the OFF vs. ON-dopaminergic medication state and a Montreal Cognitive Assessment (MoCA) of >23. In order to assess a mild to moderate Parkinson's disease population we set a cut-off of ≤10 years of diseased duration from the date of initial diagnosis, an OFF-medicine-state Hoehn and Yahr scale (H&Y) of ≤3 (9) and the absence of certain non-motor symptoms including dementia, postural instability and dysphagia (10, 11). The definition of constipation used in the study was a history of passage of hard and difficult to pass stools with no more than three bowel movements per week.

Subjects were required to adhere to stable treatment of their Parkinson's disease for 90 days before enrollment and throughout the study. They could not take unessential antibiotics or probiotics during the study. We screened subjects in groups of three with the same age (±5 years) to allow randomization to one of two treatment arms, two FMT to one placebo.

### Study design and oversight

Subjects were assessed at the University of Texas McGovern School of Medicine Clinical Research Unit at Memorial Hermann Hospital in Houston. Evaluation of subjects occurred at enrollment, 12 days afterwards (run-in), following 12 weeks of twice-weekly FMT treatment, and at 4-weeks, 6-, and 9-months post-treatment (total duration of study 12 months and 2 weeks). All motor assessments were done by a blinded Movement Disorders specialist (MCS) in the conventional “Off state,” defined as being “practically defined OFF” dopaminergic medicines 12 h prior to the exam, which is considered by many investigators standard practice in Parkinson's disease study group protocols (12, 13). Safety studies were examined twice and included complete blood count (CBC) with differential, complete metabolic panel (CMP), and urinalysis.

### FMT dose and treatment regimen

In the previous studies in which FMT was given to patients with Parkinson's disease, a single FMT was given without specification of donor fecal volume or weight. There are no FMT dose-response data available in Parkinson's disease to use in selecting a dose for our study. In reviewing the literature, we found that recurrent doses of FMT were required to treat chronic non-*Clostridiodes difficile* infection (non-CDI) disease (7). With our lyophilized product for patients with recurrent *Clostridiodes difficile* infection (CDI), we administer a dose of 100 g of donor feces, lyophilized to 1.5 g of powder, contained in 10 capsules. Not knowing tolerability of patients with Parkinson's disease for FMT, we wanted to keep individual doses below that for patients with CDI since we were giving 24 doses of product. We elected in this pilot study to give 60 g of donor feces twice a week for 12 weeks (24 doses), a dose that was approved by our Data Safety and Monitoring Board and by our University IRB. Four thoroughly screened donors provided all FMT product. Lyophilized powder derived from donor stools was packed into acid-resistant capsule shells (14) in a licensed compounding pharmacy. The lyophilized product was cultured quantitatively under anaerobic conditions and was required to meet potency assay standards required by the U.S. FDA.

At the end of the 2-week run-in period, the 12 subjects were randomly assigned to receive oral capsules of study drug (FMT) (*n* = 8) or placebo matching in appearance (*n* = 4), twice a week for 12 weeks (24 doses each).

Two capsules from each of three donors (a total of six capsules) were combined for treatment of each of the first seven subjects randomized to FMT. For the eight subject, on request of the FDA, the last two FMT doses, capsules from one of two donors were given sequentially for the remaining two treatments. The first dose of FMT or placebo was administered in the clinic for all subjects, and thereafter, capsules supplied in the clinic were taken on their own.

### Subject monitoring

Stool specimens were provided by study subjects for microbiome examination before treatment, at week 6 (mid-treatment), week 13 (1 week after treatment), and at 4, 6, and 9 months after completing treatment and were stored at −80°C.

After enrollment, at month 4, month 9, and with any early termination, subjects completed five questionnaires: the Geriatric Depression Scale Short Form (GDS-SF) (15); Parkinson's Anxiety Scale (PAS) (16); Parkinson's Disease Questionnaire (PDQ-39) (17); Parkinson's Disease Non-motor Symptoms Scale (PD NMS) (18); University of Pennsylvania Smell Identification Test (UPSIT) (19) and clinical assessment was performed using: Unified Parkinson's Disease Rating Scale (UPDRS) (20); modified H&Y (9); modified Schwab and England Activities of Daily Living Scale (ADL) (21); and REM sleep Behavior Disorder (RBD) Single Question Screening (22).

At three time points, subjects were asked to indicate their subjective improvement in Parkinson's disease-related symptoms compared to their pre-treatment level, looking at constipation, falls, sleep disturbance, reduced smell, motor deficits, and overall Parkinson's disease symptoms, scoring the symptom relief using a 100 point visual analog scale developed for this study (23), considering their pre-treatment value at 100.

An Advisory Committee reviewed the study details and protocol development and a Data Safety Monitoring Board (DSMB) reviewed the study before, during, and after study completion to assure the safety of study participants.

### Microbiome characterization

Participants sent stool samples to the University of Texas Enteric Research Laboratory (UTERL) on wet ice in multiple containers by single-day delivery, a method consistent with adequate study of microbiota (24). The UTERL is certified by Clinical Laboratory Improvement Amendments (CLIA) and the College of American Pathologists (CAP) for work done there. The samples were stored at −80°C until analyzed. Whole Metagenome Shotgun sequencing of stool samples was conducted at the Baylor College of Medicine Center for Metagenomics and Microbiome Research (CMMR) (25). Using procedures validated in the CMMR, total genomic DNA was extracted using the MagAttract PowerSoil DNA kit (Qiagen). DNA quality was determined by an automated PicoGreen assay. Individual libraries (Kapa Biosystems) were constructed from each sample and sequenced on a NovaSeq 6000 (Illumina) using the 2 × 150 bp paired-end read protocol. Bowtie2 v2.3.4.3 (26) was used to remove contaminating host reads and subsequently align microbial sequences to the MetaPhlAn3 (27) marker gene database for species classification.

### Motility studies

During the run-in period and at the 13-week clinic visit, subjects were asked to swallow a SmartPill wireless-capsule using the manufacturer's procedures to measure gastrointestinal pressures and pH (28) and to allow measurement of gut transit times and chronotropic and ionotropic motility index by counting amplitude and number of gut contractions (29, 30).

### Study blinding

Only the laboratory director (ZDJ) was aware of the source and type of product assigned to subjects. Under her supervision, capsules prepared in the laboratory and identified by number were taken to the University of Texas McGovern School of Medicine NIH supported Clinical Research Unit at Memorial Hermann Hospital System in Houston for product administration.

### Randomization

Treatments were randomly assigned to consecutive numbers by using an allocation ratio of 2:1 for treatment group and placebo group. The systematic randomization allocation method was used to develop the random assignment of study groups (31). Randomization was performed by the laboratory director (ZDJ) and was not available to other team members.

### Sample size and statistical methods

Without data on the safety and tolerability of a 24 dose, 12-week course of FMT in patients with Parkinson's disease, we powered the pilot study only for effect on constipation. Two studies in which a single FMT was administered to patients with Parkinson's disease, provide evidence that FMT should improve constipation at a rate of ~90% based on rates of 11 of 11 (100%) (32) and 5 of 6 (83%) (33) treated subjects. Assuming a rate of improvement in the placebo group of 16% (34) an alpha of 0.05 and power of 0.8, the sample size required was 8 and 4.

Agile Toolkit for Incisive Microbial Analyses (ATIMA), a stand-alone tool for microbial data exploration, was used as an integrated solution for analyzing and visualizing microbiome data (https://atima.research.bcm.edu). ATIMA2 utilizes PERMANOVA to analyze differences in overall community composition (beta diversity), while comparisons of community dispersion were determined via the Mann-Whitney U-test. Alpha diversity analyses were performed using the Mann-Whitney U-test or Kruskall-Wallace H-test as appropriate. Differentially abundant taxa by placebo or FMT treatment status were determined via LEfSe (35) using the Galaxy web platform with parameters of alpha of 0.05 and threshold of logarithmic linear discriminative analysis (LDA) score of 2.0 (36). Analyses using LEfSe were limited to taxa present in at least 30% of samples.

For the clinical symptomatic improvement, the Rasch analysis was used to convert ordinal data to interval data prior to data interpretation (37). All data were downloaded from Redcap as a comma-separated values (CSV) file and converted to the Statistical Analysis System software (SAS version 9.4; SAS Institute, Cary, NC).

Because the severity of the two groups was not matched, no clinical comparisons between groups were made in the study, and no *p*-values were calculated between treatment groups except for changes in microbiome, improvement in constipation, and motility measurements.

## Results

### Patient disposition and characteristics

**Figure 1:**
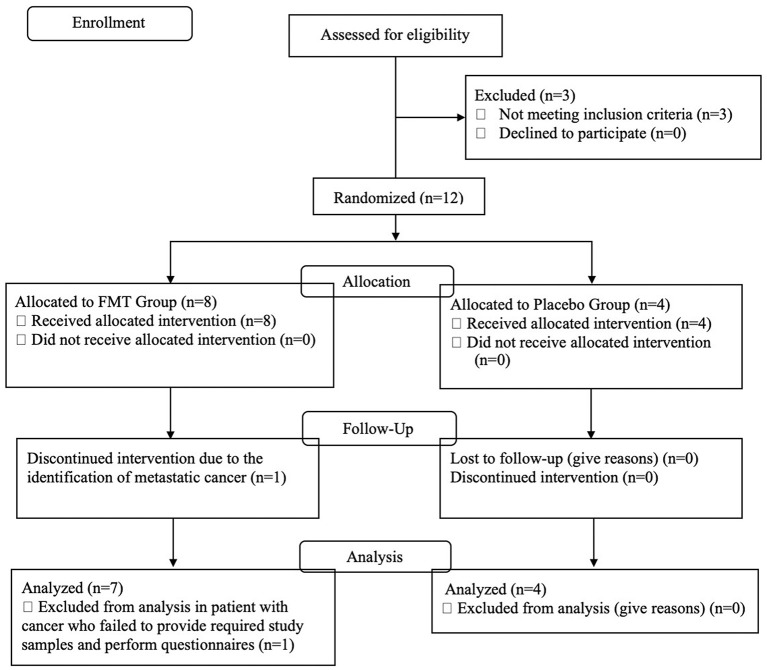
CONSORT flow diagram describing the study population, number screened, excluded, and enrolled into active treatment or placebo study arms.

Figure 1 is a CONSORT flow diagram of evaluated, enrolled, and analyzed subjects. Three of the 15 subjects failed screening eligibility. After taking eight doses of the FMT, one subject was hospitalized and found to have pre-existent, non-treatable metastatic cancer. This subject did not provide post-treatment stools or questionnaires. The DSMB declared there was no relationship between her cancer and the study drug and recommended removal from the study efficacy evaluation.

Between June 5, 2019, and November 30, 2020, the study was carried out by the Kelsey Research Foundation and the University of Texas Health Science Center. In Table 1, demographic and baseline characteristics of the study populations are provided. The FMT vs. Placebo groups differed by UPDRS total and UPSIT. Since the groups were clinically different, clinical neurologic treatment responses were not compared between treatment groups. All subjects used dopaminergic medications before baseline evaluation.

**Table 1:** Demographics and baseline values for examinations, questionnaires, and clinical findings of the two study population.

**Variable**	**Subjects randomized to FMT treatment (*****N*** = **8)**		**Subjects randomized to placebo treatment (*****N*** = **4)**	
	**Median**	**Range**	**Median**	**Range**
Gender: female to male	3:5		0:4	
Median age (years)	68.5	(61–75)	68	(56–74)
Duration Parkinson's (years)	2 (1–3)		3 (2–10)	
**Race**				
Caucasian	6 (75%)		4 (100%)	
Hispanic	2 (25%)		0	
Montreal Cognitive Assessment	28	(28–30)	27.5	(26–29)
University of Pennsylvania Smell Identification Test Assessment	22	(15–34)	13.5	10–17
Unified Parkinson's Disease Rating Scale Motor Section III	16	(13–21)	26	(18—42)
Unified Parkinson's Disease Rating Scale Total Score	24	(19–32)	47	(26–69)
Modified Hoehn and Yahr staging	1	(1–2)	2.2	(1–2.5)
Median levodopa equivalent daily dosage	446	(114–900)	645	(400–792)
Levodopa challenge, % improvement	59.3	(42.9–69.2)	51.5	(38.9–61)

### Primary endpoints

#### Safety

All patients who received at least one dose of the study drug were included in the safety analysis. Adverse events were reported in 7 (88%) of FMT- and 4 (100%) in placebo-treated subjects. Gastrointestinal complaints occurred more commonly in the active treatment group and were determined to be probably related to FMT (FMT–placebo groups): bloating/flatulence 2–0, abdominal pain/cramps/discomfort 3–1, worsening constipation 1–2, diarrhea 2–0, nausea 1–0, melena 1 (in the subject withdrawn because of cancer)−0, and pre-existent gall stone symptoms 1–0. Subjects categorized the adverse events as transient and either mild (47%) or moderate (38%) in severity. No AEs were persistent, and no treatments were withheld because of them. Safety laboratories did not identify clinical abnormalities.

#### Microbiome changes with FMT therapy

**Figure 2:**
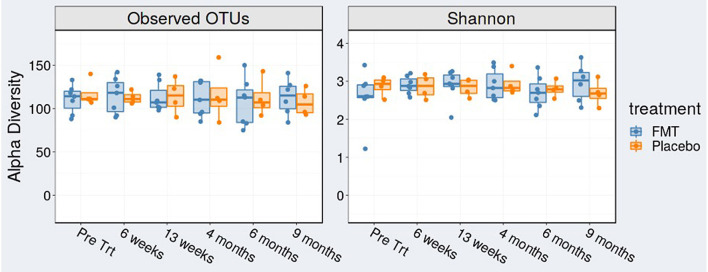
Alpha diversity [observed operational taxonomic units (OTUs)] and Shannon index at baseline (pre-treatment), at 6 weeks (mid-treatment), 13 weeks (1 week post-treatment), 4, 6, and 9 months.

We found no change in alpha diversity (richness or evenness) after examination of operational taxonomic units (OTUs) and Shannon index in FMT-treated subjects over time (Figure 2).

**Figure 3:**
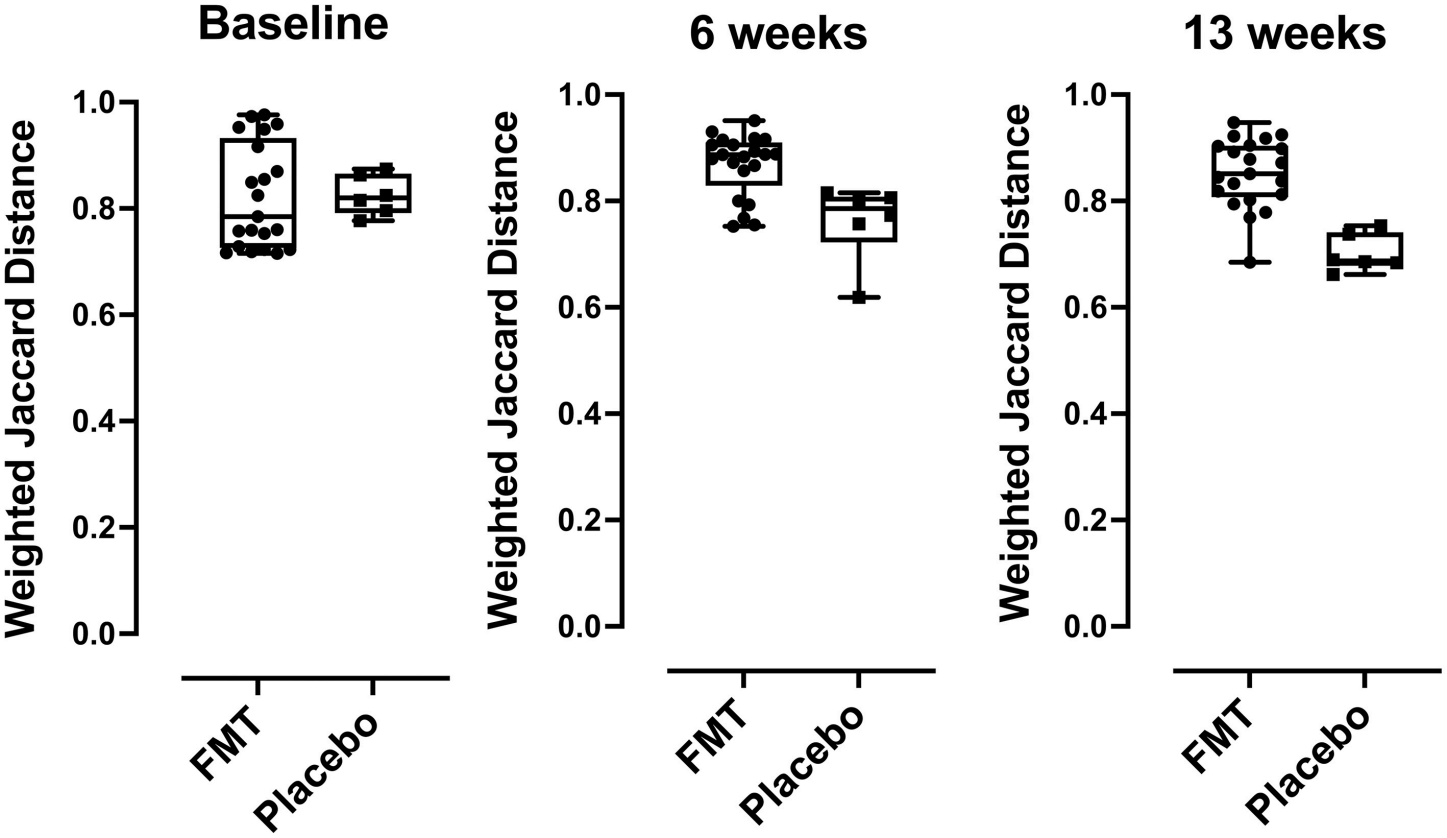
Beta diversity (taxa) differences comparing FMT- and placebo-treated subjects, comparisons of Jaccard distances between FMT and placebo recipients were compared using box plots. Over time, subjects in the placebo group had bacterial community compositions more similar to each other than those in the FMT group at baseline (*p* < 0.629), at 6 weeks *p* < 0.008, and at 13 weeks (*p* < 0.0008) post FMT. *P*-values were determined using the Mann-Whitney U-test.

Beta diversity by comparing Jaccard distance plots for the two treatment groups is provided in Figure 3. Beta diversity was similar between FMT and placebo groups at baseline (*p* = 0.629) but showed differences at 6 weeks (mid-treatment) (*p* = 0.008) and at 13 weeks (1-week post-treatment) (*p* = 0.0008).

**Figure 4:**
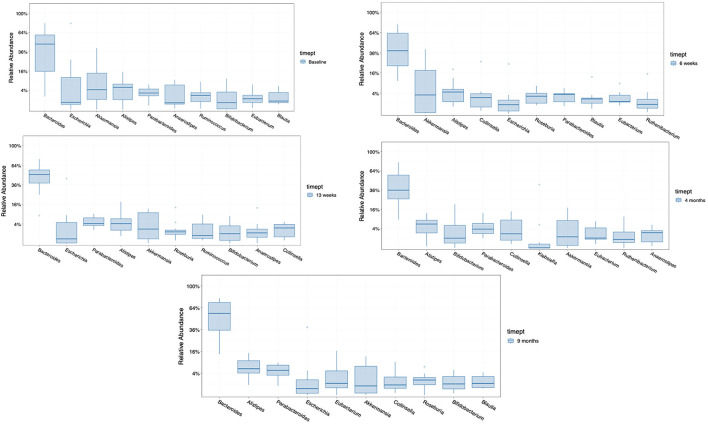
Comparison of the 10 most abundant genera in the group receiving FMT, at baseline, at 6 weeks (med-therapy), at 13 weeks (1 week after completing therapy), and at 4 and 9 months (1–6 months after completing therapy).

The ten most abundant bacterial genera found in the seven patients randomized to FMT was examined prior to beginning treatment, during 12 weeks of treatment (at week 6), after completing FMT therapy (at week 13), and at later timepoints (at month 4 and month 9) (Figure 4). The taxa that became dominant during therapy not found among the most prevalent microbiota at baseline in the group receiving FMT were *Roseburia* and *Colinsella*. These taxa persisted as dominant genera after treatment at month 4 or month 9 (1 and 6 months after completing FMT treatment). Taxa that transiently decreased in proportion were *Blautia* and *Eubacteria*.

**Figure 5:**
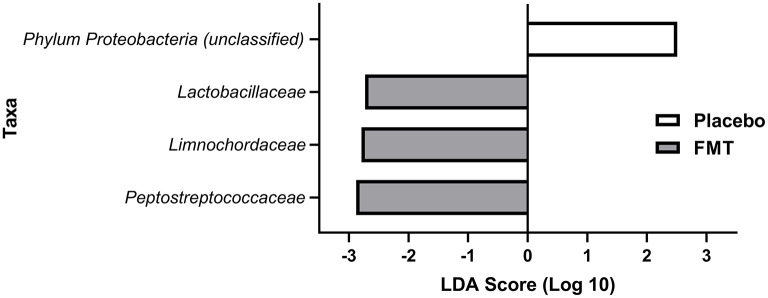
LEfSE analysis of preferentially abundant bacterial families at 13 weeks (1 week after completing treatment with either FMT or placebo) in treatment groups after eliminating families found in <30% of subjects in the treatment groups. All differences shown here at 13 weeks were statistically significant (see Results). No families were preferentially abundant in one of the treatment groups but not the other for baseline (pre-treatment) or at 6 weeks (mid-treatment).

After eliminating families present in fewer than 30% of subjects in either treatment group, there were no differences by LEfSe in preferentially abundant families across treatment groups at baseline or at 6 weeks. Three families were preferentially and significantly abundant in the FMT treatment group at 13 weeks: Lactobacillaceae (*p* = 0.038), Limnochordaceae (*p* = 0.014), and Peptostreptococcaceae (*p* = 0.008) compared with the placebo group; and with one family, Proteobacteriaceae (*p* = 0.023) was preferentially abundant in the placebo group (Figure 5).

### Exploratory efficacy end points

#### Self-reported clinical global improvement using a 100 point visual analog scale

Subjects randomized to FMT reported greater subjective clinical improvement in constipation, falls, sleep impairment, motor deficits and global Parkinson's symptoms between 4 and 16 weeks after starting treatment. Improvements after treatment were not seen with placebo treatment (Table 2).

**Table 2:** Subjective improvement in symptoms: number subjects reporting improvement and median percentile of improvement after beginning treatment compared with pre-treatment value of 100, during and after 12-weeks of oral fecal microbiota vs. placebo using a visual analog scale (0–100).

		**Constipation**		**Falls**		**Sleep disorder**		**Reduced smell**		Motor deficits^*^		**Overall Parkinson's disease**	
		**FMT**	**Placebo**	**FMT**	**Placebo**	**FMT**	**Placebo**	**FMT**	**Placebo**	**FMT**	**Placebo**	**FMT**	**Placebo**
		**(*****N*** = **7)**	**(*****N*** = **4)**	**(*****N*** = **3)**	**(*****N*** = **4)**	**(*****N*** = **6)**	**(*****N*** = **3)**	**(*****N*** = **6)**	**(*****N*** = **4)**	**(*****N*** = **7)**	**(*****N*** = **4)**	**(*****N*** = **7)**	**(*****N*** = **4)**
Four-weeks after starting FMT	Median score (0–100)	70	100	20	100	60	100	95	100	80	100	50	100
	Range	10–100	90–100	0–100	50–100	10–100	100–100	70–100	100–100	10–100	50–100	10–100	80–100
	*P*-value	<0.0001	0.2659	0.0282	0.2659	0.0002	1.000	0.0031	1.0000	<0.0001	0.2659	<0.0001	0.2659
Eight-weeks after starting FMT	Median score (0–100)	50	100	70	100	75	100	95	100	70	100	50	90
	Range	10–100	100–100	0–100	50–100	10–100	50–100	70–100	50–100	10–100	60–100	10–100	50–100
	*P*-value	<0.0001	1.0000	0.0282	0.2659	<0.0001	0.2659	0.0031	0.2659	<0.0001	0.2659	<0.0001	0.0304
Twelve-weeks last week of FMT	Median score (0–100)	20	100	0	100	50	50	95	100	70	100	50	85
	Range	10–100	50–100	0–100	50–100	10–100	0–100	70–100	0–100	10–100	80–100	10–100	0–100
	*P*-value	<0.0001	0.2659	0.0276	0.2659	<0.0001	0.0282	0.0031	0.2659	<0.0001	0.2659	<0.0001	0.0304
One-month post FMT	Median score (0–100)	30	100	0	100	45	40	95	100	70	100	50	75
	Range	10–100	50–100	0–100	0–100	20–100	00–100	70–100	0–100	30–100	80–100	10–100	0–100
	*P*-value	<0.0001	0.2659	0.0276	0.2659	<0.0001	0.0282	0.0031	0.2659	<0.0001	0.2659	<0.0001	0.0304

* Tremor, slowness of movement, limb stiffness, trouble with balance, and drooling.

#### Motor improvement

All subjects sustained motor improvement with treatment in the OFF state with greatest improvement at 1 month after treatment. At 4 months, FMT had a median reduction of 37.5% in UPDRS-Motor and 41.6% in UPDRS-Total. At 4 months placebo had a median reduction 42% in UPDRS-Motor and 38% in UPDRS-Total. At 9 months the FMT group had a median reduction of 12.5% in both UPDRS-Motor and UPDRS-Total.

Scores of standardized questionnaires GDS, PAS, NMS, and PDQ-39 and improvement of these scores with treatment are shown in Table 3. We failed to see important improvement in the questionnaires with FMT treatment.

**Table 3:** Questionnaire scores at 4-weeks and 12-weeks after randomization to FMT (*N* = 7) or placebo (*N* = 4) in subjects with Parkinson's disease failing to show improvement after 12-weeks of FMT.

**Variable**		**Run-in**		**Four-weeks after initial therapy**			**Twelve-weeks after initial therapy**		
		**Score**	**Median (range)**	**Score**	**Median (range)**	**No. of subjects with improvement (%)**	**Score**	**Median (range)**	**No. of subjects with improvement (%)**
Geriatric depression (GDS)^a^	FMT (*N* = 7)	0	1 (0–4)	0	1 (0–5)	1 (14%)	0	1 (0–5)	1 (14%)
		3		1			1		
		0		0			0		
		1		2			5		
		4		5			4		
		0		0			1		
		2		3			2		
	Placebo (*N* = 3)	1	3 (1–3)	1	3 (1–4)	0	1	3 (1–3)	0
		3		4			3		
		3		3			3		
Parkinson's Anxiety Scale (PAS)^b^	FMT (*N* = 7)	0	7 (0–14)	2	13 (2–16)	2 (29%)	0	10 (0–19)	3 (43%)
		12		16			7		
		5		2			10		
		11		13			19		
		14		16			13		
		7		14			18		
		7		3			4		
	Placebo (*N* = 3)	5	9 (5–13)	0	4 (0–11)	3 (100%)	3	5 (3–11)	3 (100%)
		13		11			11		
		9		4			5		
Non-Motor Symptoms (NMS)^c^	FMT (*N* = 7)	8	8 (4–14)	5	6 (3–10)	5 (71%)	6	6 (4–15)	5 (71%)
		7		5			4		
		6		3			4		
		10		10			15		
		12		9			10		
		4		6			6		
		14		7			9		
	Placebo (*N* = 3)	4	9 (4–12)	1	2 (67%)	11 (1–12)	2	11 (2–12)	1 (33%)
		12		11			12		
		9		12			11		
Parkinson's Disease Questionnaire (PDQ-39)^d^	FMT (*N* = 7)	13.5	40.0 (0.3–90.0)	11.5	57.7 (3.4–26.0)	6 (86%)	9.4	25.4 (6.4–100.8)	4 (57%)
		40.0		26.0			38.5		
		90.0		6.3			51.3		
		85.0		57.7			100.8		
		42.1		4.7			8.3		
		0.3		3.4			6.4		
		22.1		17.8			25.4		
	Placebo (*N* = *4*)	21.9	26 (21.9–56.7)	6.3	13 (0–20.4)	3 (75%)	15.6	17.5 (14.5–57.3)	2 (50%)
		30.0		20.4			14.5		
		16.3		19.7			19.3		
		56.7		0.0			57.3		

One subject randomized to placebo failed to complete the first three questionnaires.

a GDS, 0–5 normal; >5 depression.

b PAS, higher scores indicates great experience of anxiety.

c NMS, higher the number the higher the disease burden.

d PDQ-39, higher score indicates reduced quality of life.

### Other clinical observations after FMT

One subject randomized to FMT reported less brittle fingernails and near-total and durable remission of extensive psoriasis vulgaris of >15 years. His psoriasis prior to FMT had not shown a clinical response to clobetasol topically or systemic methotrexate according to his dermatologist.

### Gut transit times and motility changes

Due to swallowing difficulties with the SmartPill, only eight subjects were able to complete the test pre-treatment (seven were randomized to receive FMT and one placebo), and six were able to do the test post-treatment (all in the FMT group). Data for the six subjects randomized to FMT who completed both studies are provided (Table 4). Fecal microbiota transplantation treatment shortened median small bowel-, colonic-, and whole gut transit times compared with pre-treatment measurements although not significantly. The motility index, which considers the number and amplitude of contractions, was significantly improved with FMT treatment (*p* = 0.0374).

**Table 4:** Transit times and motility patterns determined by SmartPill determination pre- and post-FMT treatment in six patients.

**Gastrointestinal measurement**	**Pre-FMT**		**Post-FMT**		*P*-value^*^
	**Median**	**Range**	**Median**	**Range**	
**Gut transit times in hours**					
Gastric emptying time (normal 2–5 h)	2.77	1.34–44.59	3.31	0.5–68.58	1.0000
Small bowel transit time (normal 2–6 h)	4.33	1.15–6.59	5.20	3.52–14.36	0.1495
Colonic transit time (normal 10–59 h)	67.75	34.31–90.38	40.81	14.49–121.27	0.1495
Small bowel plus colonic transient time	70.91	35.46–95.38	47.70	18.41–126.01	0.1495
Whole gut transit (normal 10–73 h)	82.40	67.49–116.52	54.36	23.38–128.02	0.2623
**Pressure measurements**					
Frequency of small bowel contractions	1.51	0.5–4.85	5.28	0.5–8.87	0.0921
Amplitude of small bowel contractions	17.42	15.03–28.07	20.37	12.75–27.55	0.3367
Motility index+	80.22	11.31–141.59	209.03	13.38–720.44	0.0374

* Comparisons made for medians using Kruskal-Wallis test.

## Discussion

In the present study, FMT was well-tolerated by study subjects. Self-limited and non-severe gastrointestinal complaints were common but in no cases was treatment withheld, and no subjects refused a dose of FMT capsules because of these.

In a previous study, beta diversity analyses of overall gut microbiota showed a clear difference between microbial communities in Parkinson's disease and healthy controls (38). In Parkinson's disease, the intestinal microbiota have been shown to be less diverse with more clustering of strains compared with healthy controls with more diverse microbiota (39). The intestinal microbiota have been shown to be associated with motor and non-motor symptoms in Parkinson's disease, however the pathophysiology of these events is unclear (40).

In the present study, the Jaccard beta diversity of the microbiome in subjects with Parkinson's disease randomized to FMT was increased significantly compared with the placebo group, indicating a lower level of similarity among the microbiota (less clustering) and greater overall diversity of microbiota in the FMT-treated population. Mechanisms through which a more diverse microbiome may provide health benefits in Parkinson's disease are unknown. Health beneficial effects of FMT that have been identified in other settings, include protection of the gut barrier function (41), maintenance of improved gut motility and mood (42), elaboration of luminal short chain fatty acids that provide energy to the gut and stimulate intestinal propulsive contractions (43), reduction in colonic luminal pH (44), and reduction in strains of proinflammatory Proteobacteria (14).

A reduction in Firmicutes may be a factor contributing to the constipation in Parkinson's disease. In chronic constipation associated with a variety of disorders an increase in Bacteroidetes and a decease in species in the Firmicutes phylum has been identified (45). We identified increased proportions of the phylum, Firmicutes in the FMT-treated subjects of families compared to placebo-treated subjects.

In the present study, *Roseburia* and *Ruthenibacterium* became among the 10 most prevalent genera during and after FMT treatment, not found among the most common genera at baseline. *Roseburia* is a genus of butyrate-producing Lachnospiraceae (Firmacutes phylum). *Ruthenibacterium* spp. is a member of the Ruminococcaceae family, a family known to produce short-chain fatty acids and strengthen the gut barrier function and the body's immune system. *Ruthenibacterium* spp. have been identified as a member of the intestinal microbiota of patients with Parkinson's disease (46). Additionally, *Collinsella* became one of the most common genera after treatment. *Collinsella*, a genus of Actinomycetota (Actinobacteria), has been associated with protection against SARS-CoV-2 infection (47) and to be decreased in the gut microbiome during a weight loss program in obese patients with type 2 diabetes (48). A low fiber diet, unfriendly to a healthy microbiome, led to an increase in abundance of *Collinsella* in one study (49).

Two families in the Firmicutes phylum, known to have health benefits, Peptostreptococcaceae and Lactobacillaceae, were found to be statistically increased in proportion in the FMT group 1 week after completing 12 weeks therapy compared with the placebo group in the present study. In a study of germ-free rats, a strain of *Lactobacillus* accelerated intestinal transit while inhibiting growth of *Escherichia coli*, a principal member of proinflammatory Proteobacteria (50). Both Peptostreptococcaceae and Lactobacillaceae have the capacity to provide energy to the gut, contribute to intestinal immune reactivity, protect the gut lining from attachment of pathogens, and prevent malignant changes in the gut (51–53). Peptostreptococcaceae, prevalent in healthy people, has previously been shown to be reduced in patients with Parkinson's disease (54).

Limnochordaceae, a family of soil firmicutes, also found significantly more prevalent in the group randomized to FMT compared with the placebo group, is not normally considered part of the human microbiome and there are no data to suggest this family is associated with health benefits. Proteobacteriaceae, which were reduced in proportion in the FMT-treated group, compared with placebo group, represent unhealthy proinflammatory constituents of microbiome in Parkinson's disease and other chronic medical disorders and have been identified by some authors as the best single marker of a pathologic microbiome or dysbiosis (55, 56).

Most people with Parkinson's disease have chronic constipation, which can occur decades before the degeneration of the SN and onset of motor disturbances (57, 58). It is not known if the microbiota alterations in Parkinson's disease produce constipation (59), or the gut motility alterations of the disease primarily affect the gut microbiota (60, 61). The present study was powered to show a significant decrease in constipation with FMT. Subjects in the present study randomized to FMT showed significant improvement in subjective constipation during each treatment period compared with their baseline value suggesting the microbiome in Parkinson's disease plays a primary role in the development of constipation in these patients. We furthermore showed physiologic changes to support an anti-constipation effect of FMT through wireless recording that revealed that in the FMT group, small bowel-, colonic-, and whole-gut transit were shortened and motility index (62) improved compared with baseline. In a separate open study, FMT given once to 11 patients with Parkinson's disease improved constipation (32).

While we saw only transient objective evidence of motor improvement on physical exam in both groups, the individuals randomized to FMT reported significant levels of subjective improvement in symptoms common to Parkinson's disease by visual analog scales including global improvement in Parkinson's disease. This technology has been applied in a number of medical areas to measure subjective improvement in treatment studies (63–65). The subjective improvement seen in FMT-treated subjects underscores the importance of non-motor complaints in Parkinson's disease (66). More work is needed to determine the mechanisms whereby FMT improves subjective symptoms in Parkinson's disease.

Study limitations include the small number of patients, the relatively short duration of treatment, the lack of block randomization and subtyping in the drug group assignment. Additionally, we did not monitor or control the diet of the subjects. This was a study looking at the effect of FMT on Parkinson's disease and the associated microbiome and not a study designed to characterize the microbiome in Parkinson's. Thus, we do not consider a lack of non-Parkinson's disease control subjects in our study as a limitation. Other groups have conducted studies on the relationship between the microbiota and clinical phenotypes and progression rate in Parkinson's disease (67, 68). In the present study, subjects voluntarily withheld dopaminergic medication for 12 h, which constitutes a “practically defined off” state (13) and is used in the majority of disease-modifying treatment studies for Parkinson's disease (12). Withholding dopaminergic drugs for 24 h or even longer while giving a more accurate assessment for the off state, would not likely change the outcome in a clinical treatment trial in Parkinson's disease and for some patients would not be safe or well-tolerated.

This pilot clinical trial demonstrated that a 12-week treatment of FMT from multiple donors was safe and well-tolerated in subjects with mild to moderate Parkinson's disease. Favorable alterations in the microbiome by FMT were identified including greater diversity of microbiota and emergence of families within the phylum, Firmicutes. Objective Parkinson's-related motor deficits were temporarily improved and durable changes in quality of life, depression and anxiety, and other Parkinson's related symptomatology were not seen in standardized testing. However, constipation and other non-motor symptoms in the subjects randomized to FMT were reportedly improved. We believe the data obtained are encouraging and support plans for a future, larger, placebo-controlled clinical trial in Parkinson's disease with longer duration of treatment with FMT and where diet is optimized and monitored. Additionally, future studies should use disease severity for block randomization in order to avoid the probability of having groups that are not comparable in their disease stage. Microbial restoration in Parkinson's disease is a novel concept that could address the basic pathophysiology of this progressive disease with the potential for affecting the microbial dysbiosis-associated non-motor symptoms, the rate of disease progression and the levodopa response.

## Data availability statement

The datasets presented in this article are not readily available because of ethical and privacy restrictions. Requests to access the datasets should be directed to the corresponding author/s.

## Ethics statement

The study was approved by the University of Texas Committee for the Protection of Human Subjects (IRB number: HSC-SPH-18-0621—IRB telephone number +1-713-500-7943) and monitored by an experienced Data Safety Monitoring Board (DSMB). The patients/participants provided their written informed consent to participate in this study.

## Author contributions

HD, AA, Z-DJ, MS, MN, and the advisory committee: concept and design. HD, JS, Z-DJ, EB, HE, AD, and MS: acquisition, analysis or interpretation of data and administrative, and technical or material support. HD: drafting of the manuscript. Z-DJ, JS, EB, and HE: statistical analyses. AD, AA, and MN: obtaining funding. HD, Z-DJ, JS, and MS: supervision. All authors: critical revision of the manuscript. All authors contributed to the article and approved the submitted version.
